# Coordinating health workforce management in a devolved context: lessons from Kenya

**DOI:** 10.1186/s12960-020-00465-z

**Published:** 2020-03-30

**Authors:** Mathew Kariuki Thuku, Janet Muriuki, Ummuro Adano, Linet Oyucho, David Nelson

**Affiliations:** 1 IntraHealth International, Nairobi, Kenya; 2 IntraHealth International, Chapel Hill, NC United States of America; 3 KnowSolve Consulting, Ltd., Nairobi, Kenya

**Keywords:** Human resources for health, Decentralization, Health systems strengthening, Self-reliance

## Abstract

**Introduction:**

In 2013, Kenya fully and rapidly devolved health services to 47 county governments under its new constitution. It soon became evident that the coordination mechanism to manage the health workforce at a county level was inadequate. This case study describes how Kenya created an inter-county, multi-stakeholder human resources for health (HRH) coordination framework that promotes consensus, commitment, and cooperation in devolved HR management.

**Case presentation:**

Through USAID funding, IntraHealth International built the health workforce management capacity of county governments by strengthening coordination mechanisms at the national and county levels. Informed by stakeholder mapping, Kenya’s 47 counties were grouped into nine clusters with similar geographic contexts and HRH challenges. Inter-county cluster HRH stakeholder coordination forums are hosted by a rotating county-led secretariat and meet quarterly to address gaps, share successes and challenges, and track implementation of action plans. They link to the national level for capacity building, policy formulation, HRH regulation, and provision of standards. Counties have assumed ownership of the forums and contributed about US$85000 to date toward expenses.

**Conclusions:**

As a mechanism for transforming Kenya’s national HRH agenda into action at the county level, the HRH coordination framework has been instrumental in (1) expediting development, customization, and dissemination of policies, (2) enabling national HRH officers to mentor their county counterparts, and (3) providing collaborative platforms for multiple stakeholders to resolve HRH challenges and harmonize HR practices nationwide. Successes catalyzed through the inter-county forums include hiring over 20 000 health workers to address shortages; expanding the national HR information system to all 47 counties; developing guidelines for sharing specialist providers; and establishing professionalized HRH units in all 47 counties.

Kenya has made great strides in strengthening its health system through the HRH coordination framework, which supports standardization of county health operations with national goals while enabling national policy to address HRH gaps in the counties. Transitioning to fully local funding of inter-county forums is important for sustaining progress.

## Background

**Fig. 1:**
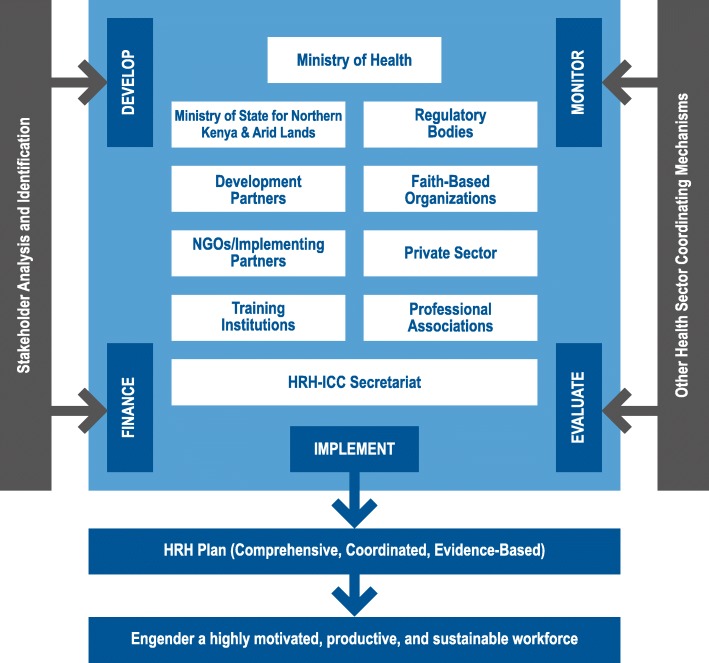
HRH-ICC coordination framework, 2012

The First Global Forum on Human Resources for Health (HRH) in 2008 raised concerns about inadequate inter-sectoral coordination for HRH planning and governance. Participating countries were asked to adopt the World Health Organization’s Country Coordination and Facilitation (CCF) [1] approach to guide HRH activities and maximize utilization of scarce resources among partners. CCF required establishing and supporting necessary governance structures for inter-sectoral coordination and collaboration to plan, implement, and monitor health workforce planning, development, and retention. In Kenya, the Ministry of Health (MOH), with support from development partners, established the National HRH Inter-agency Coordinating Committee (HRH-ICC) in February 2010 to improve health services through effective and efficient HRH planning, management, and system and policy development (Fig. 1). The MOH chaired the committee, while the membership included donors, implementing partners, health and HR regulatory bodies, medical training institutions, health sector trade unions and professional associations, the Public Service Commission, and representation from the private and faith-based sectors. The committee anchored its operations in the sector-wide approach that Kenya had adopted in 2005 to guide the coordination of health initiatives toward achieving the goals of the Kenya Health Sector Strategic Plan and Kenya Vision 2030.

### Achievements of the HRH inter-agency coordinating committee

The HRH-ICC, with technical assistance from IntraHealth International through funding from USAID, expanded its mandate to focus on the implementation of the first National HRH Strategic Plan 2009–2012. The committee established three technical working groups (TWGs)—HR management, HR development, and HR information systems—to provide input on HRH technical issues, and assigned a secretariat supported by IntraHealth. The HRH-ICC monitored implementation of the HRH strategic plan and conducted an end-term evaluation that concluded the plan’s design suitably targeted the country’s HRH needs and that it had substantially achieved its intended outcomes.

Aligned with the Country Coordination and Facilitation approach, the HRH-ICC provided a platform for developing and sustaining synergy, minimizing duplication of interventions, ensuring the focus remained on the critical HRH priorities envisioned in the national strategic plan, and leading in the coordination of a sector-wide HRH reform agenda. The HR Development TWG established a platform for coordination of pre- and in-service health training programs, contributing to completion of the first national health workforce forecast, which led to structuring of an HR development plan. The HRH-ICC also supported the MOH to map partners involved in hiring contract health workers in the public sector to harmonize their HR practices. The HRH-ICC and its partners were instrumental in developing Kenya’s 2013 HRH commitments declared during the Third Global Forum on HRH [2], and the 2014–2018 Kenya Health Sector HR strategy.

### Devolution and the need for inter-county HRH coordination

Under the Constitution of Kenya 2010, the country transitioned from a centralized system to a decentralized system in 2013, with health services fully devolved to the 47 counties. The transition took place within 6 months instead of the 3 years recommended by the MOH’s HRH transition plan [3]. Despite the formation of constitutional bodies such as the Transitional Authority, Commission for Implementation of the Constitution, Council of Governors, and Inter-governmental Coordination Committee on devolution, it soon became evident that the coordination mechanism to manage the health system at the county level was inadequate. While the HRH-ICC played an instrumental role in coordinating national HRH transition planning, there was no subnational structure for it to receive and share input with. The health workforce was also unsettled as they were unwilling to be devolved. As a result, Kenya saw several labor disputes grow into full-scale strikes affecting health outcomes in counties and, in some instances, nationally.

An HR management rapid situation analysis conducted by IntraHealth in 2014 highlighted the lack of a smooth transition to devolved workforce management, including inadequate HR guidelines, structures, and procedures; delays in salary payment, third party and statutory deductions; and pension issues, giving rise to industrial unrest. Lack of coordinated communications strained relationships between county departments of health and the national MOH. In terms of HRH capacity at the county level, the analysis found insufficient HR management within health departments; limited access to HR policies and guidelines and lack of a repository to host the policies; a weak HR regulatory framework with HR functions being managed by non-HR professionals; weak coordination mechanisms within the departments responsible for HR functions; and inadequate HR practices in areas including recruitment, performance management, disciplinary and grievance procedures, and employee relations. Undefined reporting structures led to conflicts related to roles among the various levels of leadership in the county health departments [4].

## Case presentation

**Fig. 2:**
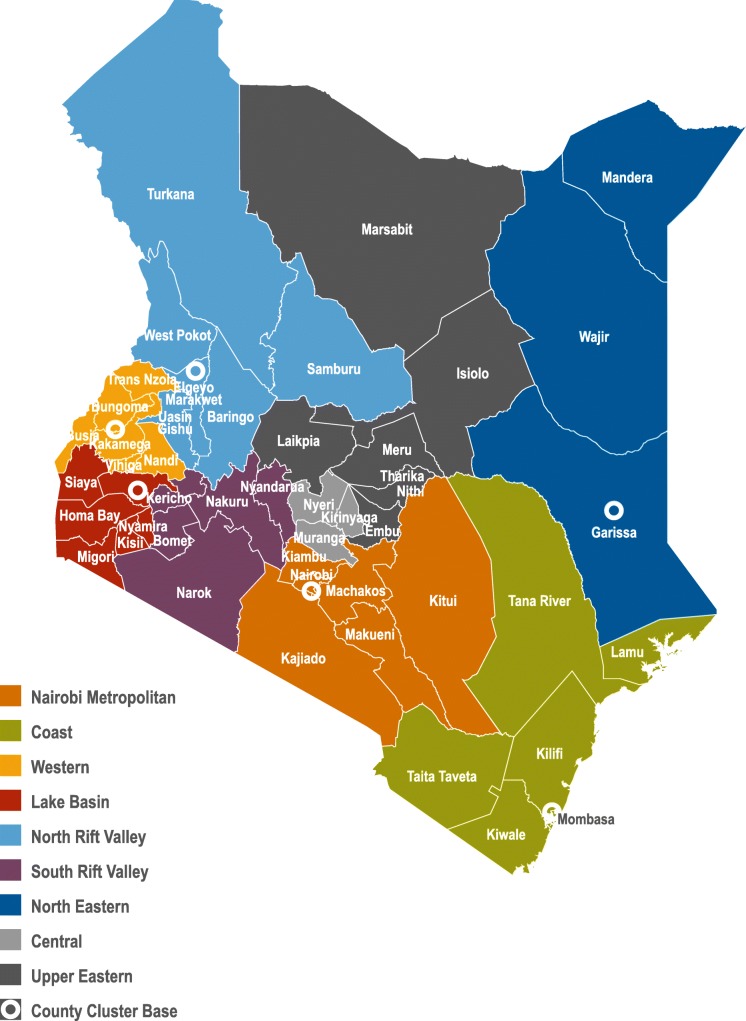
HRH county clusters

Through USAID funding, IntraHealth built the HR management capacity of county public service boards and county health departments by strengthening coordination mechanisms at national and county levels, interfacing with the Council of Governors and other constitutional bodies. The national HRH-ICC framework was cascaded to the counties beginning in 2014 through forming inter-county cluster HRH stakeholder coordination forums to address the HRH coordination gaps and to create a link to the national level for capacity building, policy formulation, HRH regulation, and provision of standards. Informed by stakeholder mapping conducted by IntraHealth and based on former administrative structures, the 47 counties were grouped into nine clusters with similar geographic contexts and HRH challenges: Central, Coast, Lake Basin, Nairobi Metropolitan, North Eastern, North Rift Valley, South Rift Valley, Upper Eastern, and Western (Fig. 2).

### Structure of the devolved HRH coordination framework

To support the devolved management of health workers, the national HRH-ICC membership was revised to comprise national MOH representatives, national stakeholders for HRH oversight functions, and representatives from the inter-county cluster HRH stakeholder coordination forums. Guided by well-defined terms of reference, the inter-county forums are hosted by a rotating secretariat (county department of health) and meet quarterly to deliberate HRH issues (e.g., better rationalization of the workforce); validate and disseminate policies; share successes and challenges and track implementation of action plans; receive outcomes of HRH-ICC meetings; and share county issues with the national level. The counties also publish and share quarterly bulletins on their successes in HRH and service delivery.

Participants in the inter-county forums include county departments of health, public service boards, and directorates of HR; medical training institutions; regulatory bodies and their local branches; health workforce trade union leaders and local chapters; national training and referral hospitals; and implementing partners involved in HRH, service delivery, and training.

The key roles of these participants are based on each stakeholder’s specific mandate and thus influence the nature of the promising practices and challenges that they bring to the forums as part of the overall coordination process. County departments of health have the mandate of managing the health workforce and improving HRH systems in the devolved setup. Public service boards and directorates of HR have the responsibility for HR management and development at the county level (e.g., recruitment, promotions, succession management). As part of their role in training health workers, medical training institutions also need to understand the labor market for their trainees. Regulatory bodies share standards of practice as well as regulatory requirements. Trade unions bring the voice of their members in representing health workers’ welfare. Implementing partners support carrying out health and HRH strengthening action plans emerging from the forum.

**Fig. 3:**
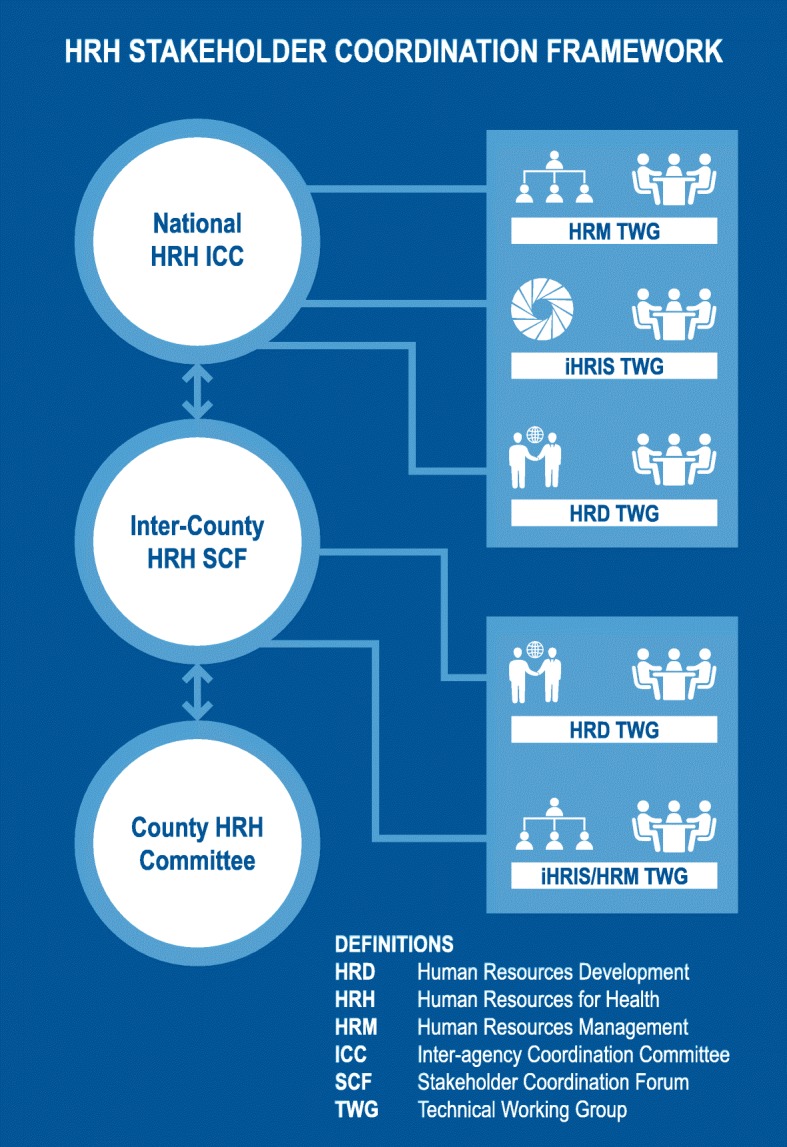
HRH stakeholder coordination framework

The forums also have TWGs for HR management and information systems and HR development that discuss key challenges and make recommendations for implementation by the cluster counties (Fig. 3). Through the information system TWGs, counties share best practices on implementing the national integrated HR information system (iHRIS). Counties can engage “iHRIS champions” from neighboring counties for direct technical support as needed, thus avoiding “reinventing the wheel.” In addition, social media (WhatsApp, Facebook) is used for peer-to-peer communication and sharing.

While the implementation of the HRH coordination framework has been led by IntraHealth with USAID funding, the framework provided from the onset a co-funding mechanism that allows counties to meet certain costs of the forums (e.g., conference space, logistics, per-diem). The rotating secretariat function ensures individual counties are not at a disadvantage cost-wise. This hosting structure has gained momentum with counties taking over the ownership and management of the forums, funding/co-funding meetings, coordinating partner support, and chairing the sessions. To date, counties have contributed about US$85000 toward hosting the forums and meeting participants’ logistical and allowance expenses.

Another coordination mechanism that has emerged from the inter-county forums is the county Chief Officers of Health Forum, which meets quarterly and is institutionalized through the Council of Governors. Deliberations of the Chief Officers of Health Forum are processed through the inter-governmental forum that brings county ministers of health and county executive committee members for health together with the Cabinet Secretary for Health (Minister of Health) and other national stakeholders. The inter-governmental forum then shares resolutions with the summit chaired by the President of Kenya that brings county governors together to resolve matters arising from the lower-level decision-making structures.

## Discussion and conclusions

As a mechanism for transforming Kenya’s national HRH agenda into action at the county level, the HRH coordination framework has been instrumental in (1) expediting the development, customization, and dissemination of policies, especially at the county level, (2) enabling national HRH officers to mentor and coach their county counterparts to provide the required stewardship at the subnational levels, and (3) providing vibrant and effective collaborative platforms for multiple stakeholders to resolve HRH challenges and harmonize HR practices nationwide.

The linkage between the national HRH-ICC and county governments has cultivated more consultation and collaboration in addressing HRH challenges. This has led to exchange of knowledge and experiences across interdependent departments, enhancing the efficiency of the health system. Peer-to-peer learning has catalyzed change and promoted harmonized understanding of policies across counties and implementation of HRH priorities including performance management for quality service provision.

The inter-county HRH forums have served to effectively engage counties to identify and discuss solutions to key HRH challenges in the context of devolution. Some of the successes supported through these forums include the following:

### County-level coordination

- Counties have hired 21 481 health workers (46% growth) over the past 6 years to address shortages and bridge gaps. In addition, 764 (98%) of contract health workers have been transitioned from partner support to counties. The forums contributed to these gains by analyzing data on workforce gaps, advocating for budgets through the county health departments and county assemblies, and developing plans to hire priority health workers.
- IntraHealth, through USAID projects, has directly supported 27 priority high HIV-burden counties to implement its open source HR information system (iHRIS) to manage data needed to make informed HRH decisions. More than 63 000 public health worker records are now automated nationwide and each county has an iHRIS dashboard with visual workforce reports.

### County cluster-level coordination

- TWGs have developed concept notes to address key HRH issues affecting counties such as the lack of specialized health workers like oncologists, family health physicians, radiologists, and other medical consultants, leading to “bottom up” policy development resulting in a guideline on sharing of specialist providers in the health sector. The guideline provides a framework for accessing specialists from other counties, national government, teaching and referral hospitals, the private sector, and faith-based organizations.
- An inter-county HR management capacity building plan has been implemented through training and mentorship. This has resulted in 1136 health sector managers and leaders with an HR mandate trained in HR management skills; 612 mentored, including 77 HR officers; and 47 county HRH units established, with 121 HR officers deployed to provide professional HR support to county health workers; 130 health sector leaders and 96 trade union leaders have also received training in leadership, management, and governance.
- The inter-county forums and information system TWG have been catalysts for peer mentorship and training to expand iHRIS to the remaining 22 counties.
- The incorporation of trade union leaders in the inter-county forums has provided an avenue for joint problem-solving and amicable resolution of disputes to avert health worker unrest.

### National-level coordination

- County stakeholders have participated in national HRH policy and guideline development through a defined process that allows customization at the county level for adoption and implementation. The forums have disseminated at least 104 policies and guidelines, including schemes of service, to all 47 counties, and over 35 policy guidelines have been developed.

Key challenges have included (1) high turnover of leadership in county and national government, requiring regular orientation of new leaders to the coordination structure; (2) county public service boards lack of full understanding of the needs of the health sector, and especially HRH, even though health makes up 60–70% of the workforce in the counties and a similarly large budget; and (3) unavailability of funders besides USAID to support the coordination mechanism, resulting in unequal attention to arid and semi-arid land counties. Donor changes in geographic scope in 2015 slowed progress toward sustainability; however, this challenge also became an opportunity for counties’ greater self-reliance in convening and co-funding meetings.

Kenya has made great strides in strengthening its health system through the HRH coordination framework. Through enhanced linkages between the national MOH and county health departments, the national Public Service Commission and county public service boards, and between the county public service boards and county health departments, it supports standardization of county health operations with national HRH goals while enabling national policy to address existing HRH gaps in the counties. Sustaining the inter-county cluster HRH stakeholders’ forums is critical if counties are to continue collaborating to effectively address the myriad of challenges that emerge in the devolved county health departments regarding health workforce management. Hence, it is key to maintain progress in establishing a county-led HRH stakeholders’ strategy with a secretariat that will manage it, and in transitioning from fully donor-supported funding to a donor/county cost-sharing structure to purely county-supported funding.

Kenya’s HRH coordination framework presents a model for creating a multi-stakeholder platform that fosters open dialog and promotes consensus, commitment, and cooperation on HRH priorities in devolved health system management. It also provides a platform for discussions on HRH strategies for implementation of the journey to self-reliance. While many solutions and recommendations have been successfully integrated into on-going HRH interventions with commendable results, the endeavor needs to be sustained over the long-term to significantly impact health care service delivery in Kenya.

## Data Availability

Data sharing is not applicable to this article as no data sets were generated or analyzed specifically for the purposes of this case study. Sources of the data cited in the article are the Kenya HRH Strategic Plan, 2019–2023 (final draft 2); Kenya’s integrated human resource information system (iHRIS); technical briefs and training reports (unpublished) of the USAID HRH Capacity Bridge project; and training and programmatic reports (unpublished) of the USAID HRH Kenya Mechanism.

## Abbreviations

CCF: Country Coordination and Facilitation
HR: Human resources
HRH: Human resources for health
HRH-ICC: Human Resources for Health Inter-agency Coordinating Committee
MOH: Ministry of Health
TWG: Technical working group
USAID: United States Agency for International Development
